# A Guide to Basic RNA Sequencing Data Processing and Transcriptomic Analysis

**DOI:** 10.21769/BioProtoc.5295

**Published:** 2025-05-05

**Authors:** Rowayna Shouib, Gary Eitzen, Rineke Steenbergen

**Affiliations:** 1 Faculty of Biotechnology, October University for Modern Sciences and Arts (MSA), Giza, Egypt; 2 Department of Cell Biology, University of Alberta, Edmonton, AB, Canada

**Keywords:** RNA-Seq, Next-generation sequencing (NGS), mRNA, Bioinformatics, Transcriptomic analysis

## Abstract

RNA sequencing (RNA-Seq) has transformed transcriptomic research, enabling researchers to perform large-scale inspection of mRNA levels in living cells. With the growing applicability of this technique to many scientific investigations, the analysis of next-generation sequencing (NGS) data becomes an important yet challenging task, especially for researchers without a bioinformatics background. This protocol offers a beginner-friendly step-by-step guide to analyze NGS data (starting from raw .fastq files), providing the required codes with an explanation of the different steps and software used. We outline a computational workflow that includes quality control, trimming of reads, read alignment to the genome, and gene quantification, ultimately enabling researchers to identify differentially expressed genes and gain insights on mRNA levels. Multiple approaches to visualize this data using statistical and graphical tools in R are also described, allowing the generation of heatmaps and volcano plots to represent genes and gene sets of interest.

Key features

• Provides a beginner-friendly protocol for RNA-Seq analysis to obtain insights into gene expression.

• Pipeline starts with raw .fastq files and involves analysis in command line/terminal and R (via RStudio).

• Yields a variety of output files that represent mRNA levels amongst different samples. Output files include count files, heatmaps, ordered lists of DEGs, and volcano plots.

## Graphical overview


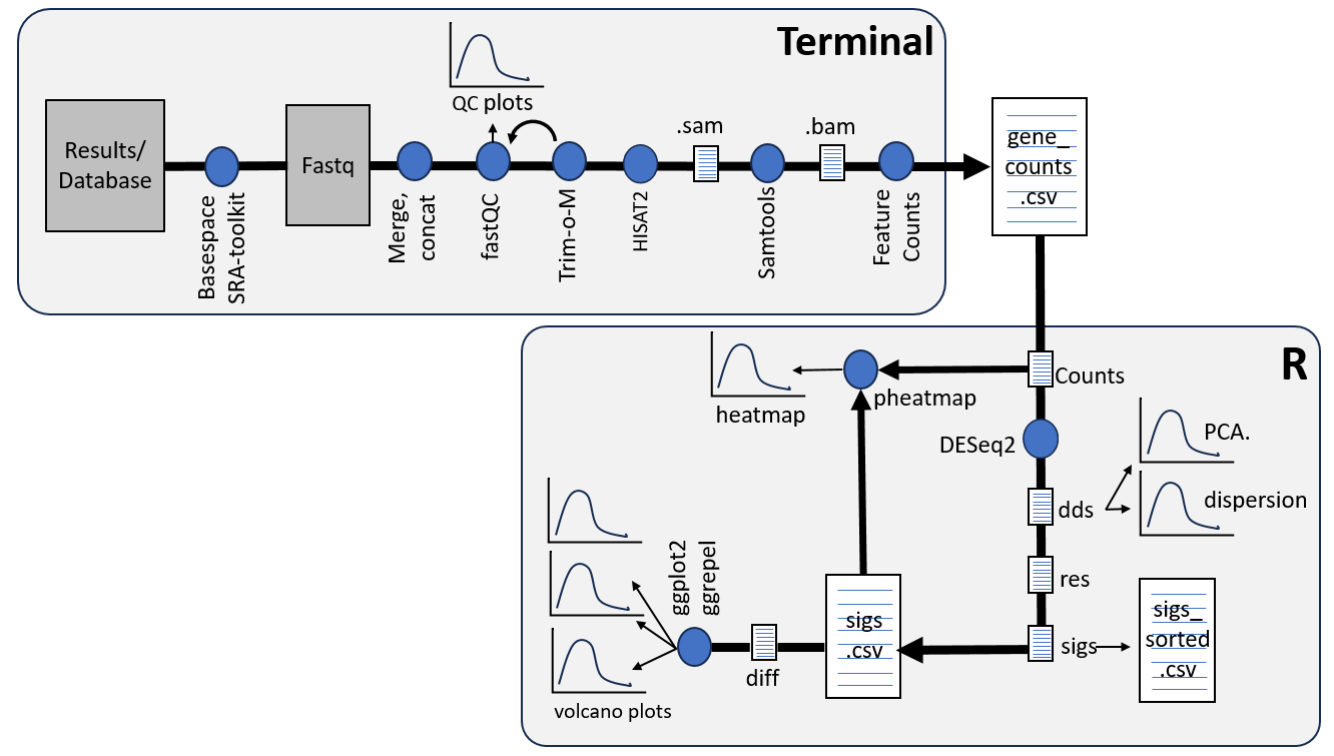


**RNA-Seq workflow.** Terminal (upper panel) is used to run the indicated programs with the output file (gene_counts.cvs) indicating the counts of each gene in a given sample. R (lower panel) run via RStudio provides statistical analysis of the counts. Details of the Terminal and RStudio scripts and the application of each program are described in the text. The blue dots represent the different software used and the arrows indicate the sequence of analysis. Furthermore, the different data outputs are shown as paper-like icons (for data tables) or graph-like icons (for graphical representations/figures of the data).

## Background

To understand the functional importance of genes of interest, biologists have turned to the analysis of the whole genome transcriptome, which involves quantifying the total mRNA content of a cell. In recent years, omics technologies have offered a novel approach to obtaining high-throughput data in biological systems in response to different conditions and experimental treatments. One such technology, RNA sequencing (RNA-Seq), involves the utilization of next-generation sequencing (NGS) and allows for large-scale examination of the RNA content of cells [1]. This has allowed for its widespread use to assess gene expression patterns and also for the identification of novel RNA transcripts.

In transcriptomic RNA-Seq—the focus here—the mRNA content of cells is examined quantitatively through the specific selection of mRNA poly(A) tails or through the depletion of ribosomal RNA (rRNA). This is followed by reverse transcription of the mRNA into cDNA. Every cDNA synthesized subsequently gets sequenced by short NGS reads, and the number of reads per transcript gets quantified. We used the workflow presented in the graphical overview to analyze RNA-Seq data obtained from stimulated airway epithelial cells [2]. For this study, gene signatures related to inflammation and cellular trafficking were the specific analyses of interest, but the pipeline is amenable to many explorations of differential gene expression in response to different treatments and perturbations.

Output from NGS platforms is typically in the format of FASTQ files, which consist of sequenced reads. These reads must be mapped or aligned to the reference genome of the samples, which may be performed using software such as HISAT2, STAR, or TopHat. The pipeline described in this method uses HISAT2 for alignment. Information on the counts of each transcript can be obtained using featureCounts software. This can then be followed by differentially expressed genes (DEG) analysis and plotting of gene expression data. All of these steps require a computational pipeline, which is described here in detail for DESeq2. The first part of this computational pipeline is performed in Terminal, also known as Shell, which runs code from the command line, ideally using a high-capacity computer for faster data processing. Then, DEG analysis is performed in RStudio. Note that coding in Terminal and RStudio is case-sensitive, and slight differences in code spacing or capitalization can render it invalid.

In this protocol, RNA quality control and library preparation steps performed prior to NGS are not discussed; rather, a straightforward computational pipeline to analyze RNA-Seq data is provided. Specifically, detailed steps are outlined that allow researchers with little to no previous expertise with RNA-Seq data analysis or bioinformatics to go from raw FASTQ-format files obtained from NGS sequencing, or obtained from online databases, to information on differential gene expression levels and different modes to present such data such as volcano plots and heatmaps.

An example analysis using data from the GEO is provided as Supplementary Information.

## Software and datasets

1. Conda

2. FastQC [3]

3. Trimmomatic [4]

4. HISAT2 [5]

5. Samtools [6]

6. featureCounts (part of the Subread package) [7]

7. R and R Studio (https://cran.r-project.org/web/packages/litteR/vignettes/litteR-installation.html)

8. Bioconductor (R package) [8]

9. pheatmap package (R package) [9]

10. ggplot2 (R package) [10] and ggrepel (R package) [11]

## Procedure

## STEP 0: Software installation

Start by processing FASTQ files for quality control and further analysis of sequencing information in Terminal. Terminal is a text interface where files can be downloaded, folders created, and software installed and run using text commands. It may also be referred to as the Linux command line or Shell. In this protocol, the first phase of the analysis will be carried out using different software performed in Terminal (**steps 0–7**), after which the analysis will transition to RStudio (**steps 8–12**). The following link provides a resource as a primer on Terminal to understand how to create files and understand commands: https://ubuntu.com/tutorials/command-line-for-beginners#1-overview.

Throughout this protocol, sample codes are provided that can be adapted to the user’s own files and folder locations. Hashtag symbols (#) denote explanations or commentaries, and any text preceded by a # is not processed as a command by Terminal or RStudio.

To install appropriate software or packages, download the Bioconda package manager. Bioconda or Conda allows the installation of packages such as fastqc (used for quality control) and trimmomatic, which will be used to trim off adapter sequences and short reads of poor quality. There are a variety of different Conda formats, but in this protocol, Miniconda will be used, which can be installed and activated using:

https://conda.io/projects/conda/en/latest/user-guide/install/index.html.

Following installation, restart Terminal.

Load the Terminal app (this can be done from the start menu by typing *Terminal*).

Use the following code in Terminal to verify Conda has been installed:

conda --version

# verify conda is installed.

conda update conda

# update conda to latest version.

y

# yes when asked to proceed ([y] or n).

Use the following code in Terminal to install fastqc, trimmomatic, HISAT2, Samtools, and subread (a package that includes featureCounts tool) software:

conda install -y -c bioconda fastqc trimmomatic hisat2 samtools subread

If prompted, an updated version of libggc may be needed. This and other packages can be found at https://anaconda.org/conda-forge/libgcc-ng, or it can be downloaded by running this script:

conda install -c conda-forge libgcc-ng

## STEP 1: Download and prepare FASTQ files

Depending on the sequencing platform used, FASTQ files may need to be downloaded from a special downloader. For instance, a software called Basespace is the typical Illumina downloader, which can be downloaded from: https://developer.basespace.illumina.com/docs/content/documentation/cli/cli-overview. Data deposited in the Gene Expression Omnibus (GEO) databases (https://www.ncbi.nlm.nih.gov/geo/) can be downloaded with the SRA toolkit, which can be downloaded from: https://github.com/ncbi/sra-tools/wiki/02.-Installing-SRA-Toolkit.

It is recommended that all FASTQ files be placed in a specific folder on the computer where Terminal is going to be used (e.g., a folder named FASTQ). A new folder can be made manually or using the *make directory* or *mkdir* command in Terminal. Once this folder is created, the raw files can be manually moved into that folder. After this, the working directory of Terminal will need to be changed to that folder so that these files can be accessed and processed. This can be done by using the *change directory* or *cd* command in Terminal. Simply type “cd” followed by the folder path. The folder path can be identified through copying that folder manually as regular and pasting in Terminal:

mkdir FASTQ

# makes a new folder titled ‘FASTQ’.

# once this is complete, manually move raw .fastq files into the ‘FASTQ’ folder

cd FASTQ

# to change the working directory to ‘FASTQ’ folder.

# download FASTQ files from browser or downloader and manually move into the working folder (e.g. FASTQ folder)

# A typical folder path in Terminal for Linux or Mac users may look like the following: C:/Users/[user name]/Documents/FASTQ

# note that folder paths in Windows may employ the backslash instead of the forward slash used throughout this protocol. The backslash is used in this protocol to split long commands onto multiple lines.

Verify that all the files are present in the folder by using the *ls* command:

ls

# this will list all the files present in the ‘FASTQ’ folder.

Files may then need to be unzipped, for example, if they end in a .gz format. This can be done using the *gunzip* command:

gunzip *type file name*

# depending on file size, this may take a while to run. It will be completed when the unzipped file (that no longer ends in .gz) appears in the folder and when the prompt appears again in Terminal.

# repeat this for all files.

Unzipping can also be done for multiple files at the same time in a loop command. For instance, if files are named Sample 1_L001_R1.fastq.gz, Sample 1_L002_R1.fastq.gz, Sample 1_L003_R1.fastq.gz, and Sample 1_L004_R1.fastq.gz [sequences for Sample 1, read 1 (R1) spread over four lanes (L00x)], then use the following loop command:

for file in "Sample 1_L00{1..4}_R1.fastq.gz"; do

 gunzip "$file"

done

If files are named Sample_A.fastq.gz, Sample_B.fastq.gz, Sample_C.fastq.gz., etc., or Sample_1.fastq.gz, Sample_2.fastq.gz, Sample_3.fastq.gz., etc., then use the following command, adjusting for the exact file name as needed:

for file in Sample_*.fastq.gz; do

 gunzip "$file"

done

Subsequently, files may also need to be concatenated or merged. This is because a single sample may have yielded multiple files as a result of a sample being split over multiple lanes in the flow cell during sequencing. *Note: If paired-end sequencing was performed, one must concatenate files from one read (e.g., R1) together, and files from the other read (e.g., R2) together. Do not merge R1 and R2 files together. This will yield two files (R1 and R2) for each sample after merging.*

To pool data from different lanes together, use the *concatenate* or *cat* command in Terminal. For example, if R1 files are named Sample 1_L001_R1.fastq, Sample 1_L002_R1.fastq, Sample 1_L003_R1.fastq, and Sample 1_L004_R1.fastq, concatenate all R1 files using the following command:

cat Sample 1_L001_R1.fastq Sample 1_L002_R1.fastq Sample 1_L003_R1.fastq Sample 1_L004_R1.fastq > concatenated_Sample1_R1.fastq

# This concatenates/merges together the different lanes for Sample 1 Read 1 into one file named ‘concatenated_Sample1_R1.fastq’. Files are listed with a space in between them. Adjust accordingly to file names.

Then, repeat this for R2 reads for the same sample:

cat Sample 1_L001_R2.fastq Sample 1_L002_R2.fastq Sample 1_L003_R2.fastq Sample 1_L004_R2.fastq > concatenated_Sample1_R2.fastq

Repeat for remaining samples.

## STEP 2: Run quality control on FASTQ files

Following sequencing, quality control (QC) needs to be performed to assess the quality of read data, including information on base quality and the level of contamination with adapter sequences [12]. Assessing the level of contamination with adapter sequences before and after trimming those adapter sequences with the Trimmomatic tool is particularly important to determine the success of trimming. A commonly used tool for such QC is the FastQC tool [3] (https://www.bioinformatics.babraham.ac.uk/projects/fastqc/), which would have been installed with conda as described earlier.

First, in Terminal, either verify or change the directory to the folder where all the concatenated files are saved; change the directory using the *cd* command as described earlier:

pwd

# shows the current directory/folder.

cd *paste folder path*

# to change directory to correct folder if needed.

Use *ls* command to confirm concatenated files are present in that folder:

ls

# to show all files in that folder.

Next, make a new folder where all the FastQC reports will be saved. This can be done manually or using the *make directory* or *mkdir* command in Terminal as follows:

mkdir fastqc_reports

# makes a new folder where FastQC reports will be stored.

# new path would be C:/Users/[user name]/fastqc_reports.

fastqc -o fastqc_reports concatenated_Sample1_R1.fastq

# fastqc will run on concatenated_Sample1_R1 file (can be replaced with user’s file name) and will output the file into fastqc_reports folder.

fastqc -o fastqc_reports concatenated_Sample1_R2.fastq

# run again for paired end R2.

Repeat for the remaining samples.

**Figure 1.**
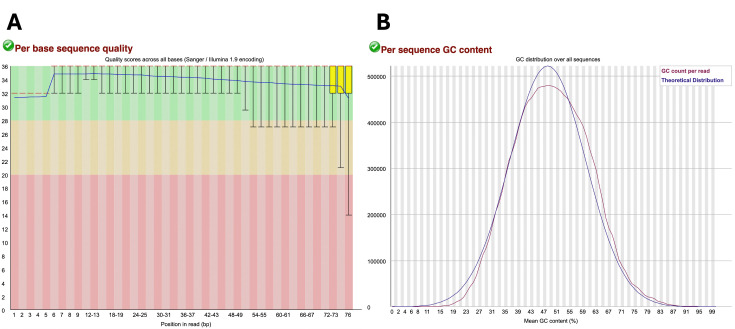
Example of sample data considered of appropriate quality. (A) “Per base sequence quality” graph with the green region indicating very good quality scores (Phred score > 28), the orange region indicating reasonable quality scores (Phred score > 20), and the red region indicating poor quality scores (Phred score 0-20). (B) “Per sequence GC content” generated in FastQC output report. The blue line represents the theoretical distribution that assumes a normal distribution around the expected GC content of the organism’s genome. The red line shows the GC content (%) vs. the number of reads of the sample being analyzed. A red line that follows similarly to that of the theoretical distribution is indicative of high-quality uncontaminated samples.

Running FastQC yields .html files that can be opened with a web browser and provide a graphical representation of the read quality for ease of assessment. The “Per base sequence quality” plot (https://www.bioinformatics.babraham.ac.uk/projects/fastqc/Help/3%20Analysis%20Modules/2%20Per%20Base%20Sequence%20Quality.html) yields a graph with the y-axis representing the quality scores and the y-axis denoting the position of each nucleotide along the read. For the y-axis, the green region indicates very good quality (Phred score > 28), the orange region indicates reasonable quality (Phred score > 20), and the red region indicates poor quality (Phred score 0-20). Reads should mostly be in the green range, especially at the beginning of the read, with reads of low quality being mostly at the end of the read **(Figure 1A)**. The “per sequence GC content” plot (https://www.bioinformatics.babraham.ac.uk/projects/fastqc/Help/3%20Analysis%20Modules/5%20Per%20Sequence%20GC%20Content.html) shows the GC content for all reads, which should have a normal bell-shaped distribution that follows the theoretical distribution curve with the peak showing the overall GC content of the organism **(Figure 1B)**. Other parameters assessed by FastQC are listed and further explained here: (https://kbase.us/applist/apps/kb_fastqc/runFastQC/release).

## STEP 3: Trim adapters and reads of low quality using Trimmomatic

As shown in **step 2**, reads may have bases of low quality, especially toward the end of the reads (see **Figure 1A**). Therefore, reads must often be trimmed or truncated to remove low-quality bases as well as adapter sequences.

During the library prep of samples, a particular kit for adapter sequences would have been used (for instance, the commonly used TruSeq2-PE.fa). Obtain the relevant sequence file for the adapters used so that they can be trimmed using Trimmomatic. The relevant sequence of adapters can typically be found in this GitHub repository: (https://github.com/usadellab/Trimmomatic/tree/main/adapters).

Once the relevant adapter sequences files are obtained, copy the sequences and paste them into a text editor (Text Editor on Unix, Notepad on Windows, or TextEdit on Mac). This can then be saved with the relevant name, e.g., TruSeq2-PE.fa, in the working folder. It is important to ensure that the file type is set to plain text or "Text Only" format. Saving the adapter sequences in these plain text formats ensures that Trimmomatic can properly read and process them. Note that Word and Google Docs are more complex files with a large amount of information and are not easily read by Trimmomatic.

Again, change to the correct folder where concatenated files are saved:

pwd

# shows the current directory/folder.

cd *paste folder path*

# to change directory to correct folder if needed.

Verify that concatenated files and the adapter sequences file are present in the folder using *ls* command:

ls

# to show all files in that folder.

Next, run the Trimmomatic tool. With single-end reads, use the following template to run Trimmomatic:

trimmomatic SE -threads 4 -phred33 \

 <input_file.fastq> \

 <output_file.trimmed.fastq> \

 ILLUMINACLIP:<adapters_file.fa>:2:30:10 \

 LEADING:3 \

 TRAILING:3 \

 SLIDINGWINDOW:4:15 \

 MINLEN:36

Using the previous file name examples and adapter sequence example, this may look like:

trimmomatic SE -threads 4 -phred33 \

 concatenated_Sample1_R1.fastq \

 Sample1_R1_trimmed.fastq \

 ILLUMINACLIP:TruSeq2-PE.fa:2:30:10 \

 LEADING:3 \

 TRAILING:3 \

 SLIDINGWINDOW:4:15 \

 MINLEN:36

If the data is paired end, there would be two input files (R1 and R2) per sample. The Terminal command may look like this:

trimmomatic PE -threads 4 -phred33 \

 concatenated_Sample1_R1.fastq concatenated_Sample1_R2.fastq \

 Sample1_R1.paired.fastq Sample1_R1.unpaired.fastq \

 Sample1_R2.paired.fastq Sample1_R2.unpaired.fastq \

 ILLUMINACLIP:TruSeq2-PE.fa:2:30:10 LEADING:3 TRAILING:3 SLIDINGWINDOW:4:15 MINLEN:36

*Note: Although Trimmomatic produces 4 output files, 2 paired and 2 unpaired, the paired output files will be used for downstream alignment in the following steps.*

Repeat for remaining samples. Alternatively, run commands for samples simultaneously to process them all at once. This can be done by separating Trimmomatic commands with the & sign and backslash \ as follows:

trimmomatic PE -threads 4 -phred33 \

 concatenated_Sample1_R1.fastq concatenated_Sample1_R2.fastq \

 Sample1_R1.paired.fastq Sample1_R1.unpaired.fastq \

 Sample1_R2.paired.fastq Sample1_R2.unpaired.fastq \

 ILLUMINACLIP:TruSeq2-PE.fa:2:30:10 LEADING:3 TRAILING:3 SLIDINGWINDOW:4:15 MINLEN:36 & \

trimmomatic PE -threads 4 -phred33 \

 concatenated_Sample2_R1.fastq concatenated_Sample2_R2.fastq \

 Sample2_R1.paired.fastq Sample2_R1.unpaired.fastq \

 Sample2_R2.paired.fastq Sample2_R2.unpaired.fastq \

 ILLUMINACLIP:TruSeq2-PE.fa:2:30:10 LEADING:3 TRAILING:3 SLIDINGWINDOW:4:15 MINLEN:36 & \

trimmomatic PE -threads 4 -phred33 \

 concatenated_Sample3_R1.fastq concatenated_Sample3_R2.fastq \

 Sample3_R1.paired.fastq Sample3_R1.unpaired.fastq \

 Sample3_R2.paired.fastq Sample3_R2.unpaired.fastq \

 ILLUMINACLIP:TruSeq2-PE.fa:2:30:10 LEADING:3 TRAILING:3 SLIDINGWINDOW:4:15 MINLEN:36 &

A thorough description of Trimmomatic parameters is provided in the manual (http://www.usadellab.org/cms/uploads/supplementary/Trimmomatic/TrimmomaticManual_V0.32.pdf) and online guide (http://www.usadellab.org/cms/?page=trimmomatic) but is also explained briefly below:

- SE/PE: single-end/paired-end data
- Backslash (\): used to indicate that code continues on the next line.
- Threads: specifies the number of threads used by the computer for processing, affecting speed.
- Phred: specifies the quality scores of the FASTQ input files
- concatenated_Sample1_R1: our input file
- Sample1_R1_trimmed.fastq: our trimmed output file
- ILLUMINACLIP: specifies the file with the relevant adapter sequences and sets clipping parameters
- LEADING: removes low-quality (<3) bases at the start of the read
- TRAILING: removes low-quality (<3) bases at the end of the read
- SLIDINGWINDOW: sets the base length of the sliding window (4 bases) and specifies the minimum average quality within window (>15)
- MINLEN: specifies minimum length of reads (>36)

## STEP 4: Run quality control again on trimmed files

mkdir fastqc_trimmed_Sample1_reports

# makes a new folder where FastQC reports for trimmed files will be stored.

fastqc -o fastqc_trimmed_Sample1_reports \

Sample1_R1.paired.fastq Sample1_R2.paired.fastq

# since subsequent alignment will use the paired trimmed outputs of Trimmomatic, run FastQC on these files here.

**Figure 2.**
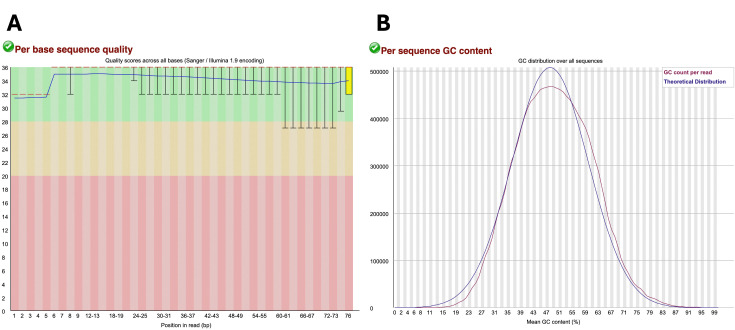
Example of post-trimming sample data considered of appropriate quality. (A) “Per base sequence quality” graph with the green region indicating very good quality scores (>28), the orange region indicating reasonable quality scores (>20), and the red region indicating poor quality scores (0–20). (B) “Per sequence GC content” generated in FastQC output report. The blue line represents the theoretical distribution, which assumes a normal distribution around the expected GC content of the organism’s genome. The red line shows the GC content (%) vs. the number of reads of the sample being analyzed. A red line that follows similarly to that of the theoretical distribution is indicative of high-quality uncontaminated samples.

Then, check the .html FastQC output files, which should now show the majority of data points on the “Per base sequence quality” graph in the green zone. That is, most of the sequences that had low quality (i.e., data points in the red and yellow regions have been eliminated), and the quality of the bases toward the end of the reads has improved **(Figure 2A)**. Per sequence, the GC content may not show large improvements following trimming **(Figure 2B)**.

## STEP 5: Align to genome using HISAT2

Now that the low-quality and adapter sequences have been trimmed from read files, reads must be aligned to corresponding genes within the genome to obtain information on the number of reads per gene, which will enable gene expression quantification.

Although multiple alignment tools exist such as STAR and TopHat, this protocol utilizes the HISAT2 software because it is considered an accurate aligner that is also quite fast compared to other aligners [13].

HISAT2 would have been previously installed as described in **step 0** using conda.

The relevant genome index file for the organism of interest must first be obtained. Genome indexes for *Homo sapiens* and a variety of commonly-used model organisms for HISAT2 alignment can be found at https://daehwankimlab.github.io/hisat2/download/#h-sapiens.

For this protocol, the human genome index (grch38) will be used as the reference genome (https://genome-idx.s3.amazonaws.com/hisat/grch38_genome.tar.gz). The downloaded genome index may need to be unzipped (often simply by opening it), which yields a folder called grch38. The grch38 folder will have multiple files with different extensions.

Next, a new folder (typically created inside the previous working folder) can be made to store HISAT2 output files:

mkdir HISAT2

# makes a new folder/directory.

Once the HISAT2 folder is created, the trimmed files (Trimmomatic output files) will need to be manually moved into that folder. The reference genome folder (grch38) will also need to be moved into the HISAT2 folder before running HISAT2.

cd *paste HISAT2 folder path*

# to change directory to correct folder if needed.

hisat2 -x grch38/genome -1 *paste trimmed R1 file path* -2 *paste trimmed R2 file path* -S Sample1_aligned.sam

#Grch38_genome.tar.gz was unzipped, which creates a series of files with “genome” as the base name. These files are referenced here as the index (specified after -x). The file paths/locations for the trimmed R1 (specified after -1) and R2 (specified after -2) files are then listed (e.g., hisat2 -x grch38/genome -1 /home/[user name]/RNASeq/HISAT2/Sample1_R1.trimmed.fastq -2 /home/[user name]/RNASeq/HISAT2/Sample1_R2.trimmed.fastq -S Sample1_aligned.sam). Lastly, the desired name of the output file is specified (after -S) with “.sam” as the extension (e.g., Sample1_aligned.sam). Repeat for the remaining samples or run simultaneously as described earlier.

## STEP 6: File conversion

The output file from HISAT2 is a tab-delimited text (.sam) file. Before proceeding with the remaining analysis, these files need to be converted to a binary (.bam) file. In addition, these files can also be sorted by genome coordinates and indexed to streamline downstream analysis [14,15]. These steps can be performed using the Samtools software [6].

First, confirm the folder/directory is the HISAT2 folder from before as it has all the required input files:

pwd

# confirms current directory.

cd *paste HISAT2 folder path*

# change directory to correct folder if needed.

Next, SAM files can be converted to BAM files, sorted, and indexed as follows:

samtools view -S -b Sample1_aligned.sam > Sample1_aligned.bam

samtools sort Sample1_aligned.bam -o Sample1_sorted_aligned.bam

samtools index Sample1_sorted_aligned.bam

Repeat for the remaining samples or run simultaneously as described earlier.

## STEP 7: Count reads per gene using featureCounts

To obtain quantitative information on the number of reads mapped to genomic *features* (i.e., genes), a tool called *featureCounts* from the *Subread* package can be used [7]. *featureCounts* exports a summarization file with quantitative values or *counts* for reads successfully assigned to genes and also outlines those that could not be assigned.

BAM files from the previous steps will be used as input for featureCounts. In addition, an annotation file that includes detailed structural and functional information on genomic features must also be supplied as input for featureCounts. This annotation file must be specific to the organism of interest.

Again, confirm the working directory is set to the HISAT2 folder as it contains all the input BAM files:

pwd

# confirms current directory.

cd *paste HISAT2 folder path*

# change directory to correct folder if needed.

Next, download the gene annotation file in the GTF format using the *wget* command as follows:

wget https://ftp.ensembl.org/pub/release-109/gtf/homo_sapiens/Homo_sapiens.GRCh38.109.gtf.gz

# this protocol used the 109 release. Check the ‘gene annotation’ tab at https://www.ensembl.org/Homo_sapiens/Info/Index for the latest releases.

Next, the downloaded GTF file may need to be unzipped. This can be performed manually by opening the folder where it is located and clicking on it to unzip it. The extracted file can then be moved to the HISAT2 folder (which contains all BAM files).

featureCounts may then be run as follows:

featureCounts -p -a Homo_sapiens.GRCh38.109.gtf -o Sample1_counts.txt \

Sample1_sorted_aligned.bam

# this command will export an output file titled ‘Sample1_counts.txt’ using ‘Sample1_sorted_aligned.bam’ as the input file.

# -p indicates paired-end data.

# -a specifies the name of the annotation file.

This outputs a .txt file that will need to be processed for simplicity. Since it is a .txt file, it can be opened with Excel. To simplify further analysis performed by R, keep only “GeneID” and “Counts or Counts per sample” columns and manually delete all remaining columns (for instance, chr. start, length, etc.) in Excel. Save this file as a .csv file.

Repeat for the remaining samples or run simultaneously.

**Figure 3.**
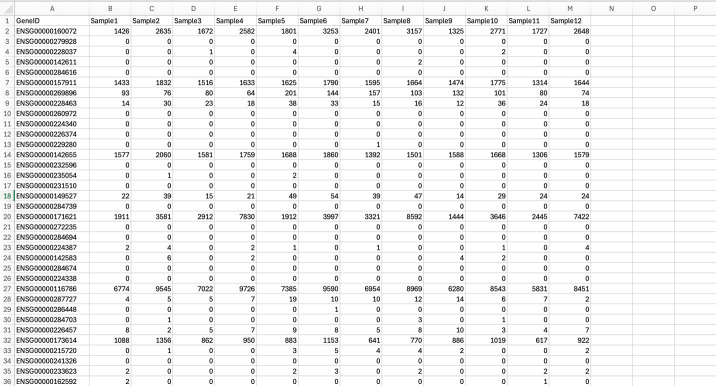
Compiling counts from different samples/replicates into one gene counts file. Columns containing gene counts from each sample file were pasted into one file.

Once count files have been obtained for all samples/groups, data columns from each sample file can be compiled together in Excel by pasting count columns next to each other in one .csv file. Note that GeneIDs will be listed in the same order as the annotation GTF file, so the same annotation file must be used for all samples to ensure that counts from different samples line up to GeneIDs correctly when copying and pasting them into one file. The compiled file may look as indicated in **Figure 3**.

The generated gene counts file can then be used as an input file for DESeq2 analysis in R for further analysis and graphical representation of the results in different formats. However, this file can also be used as an input for other analyses such as GSEA (gene set enrichment analysis) and GO (gene ontology).

## STEP 8: Run DESeq2 in RStudio

While the previous steps were all carried out in Terminal, analysis now transitions to R programming. For a more user-friendly interface, RStudio is used to run R codes. Both R and RStudio must be installed, which can be done from:

https://cran.r-project.org/web/packages/litteR/vignettes/litteR-installation.html. The following codes will allow for the analysis of differentially expressed genes (DEGs) as well as for the graphical representation of data, such as through heatmaps or volcano plots.

Codes are to be run in the Script Editor pane in RStudio (top left pane). The R package DESeq2 [16] will be used to analyze counts information obtained from featureCounts and identify DEGs. DESeq2 performs internal normalization of counts with the following resource providing a primer on this: https://hbctraining.github.io/DGE_workshop/lessons/02_DGE_count_normalization.html. DESeq2 is a tool that requires replicates for each sample to provide an analysis of gene counts. In this protocol, we illustrate the use of 3 biological replicates for each treatment group. Since there were 4 treatment groups, there are 12 *samples* or groups.

There are a number of software packages that perform statistical analyses of sequencing reads, which are used to determine DEGs. DESeq2 and EdgeR are R packages that use slightly different statistical methods to provide output tables (log fold change, p-value, false discovery rate) of differentially expressed genes from replicate samples. Other programs such as cuffdiff, which is part of the Cufflinks statistical analysis package, use fragment reads (normalization based on FPKM, fragments per kilobase per million mapped fragments) and can provide isoform-level DEG analysis [17]. All of these steps require a computational pipeline, which is described here in detail for DESeq2.

First, Bioconductor should be installed in R. Bioconductor is a collection of packages relevant to bioinformatic analysis and thereby relevant to RNA-Seq analysis. Once installed, Bioconductor packages can be managed by the BiocManager package, which allows installation and loading of different Bioconductor packages.

To download BiocManager, use the following command in RStudio. Type the command in the Script Editor pane and press run. Note that the cursor needs to be on the line that needs to be run.

install.packages("BiocManager")

# this installs the BiocManager package.

BiocManager::install()

# this installs the core packages of Bioconductor.

BiocManager::install(version = "3.15")

# this command can be used to update BiocManager to the latest version if already installed. Replace “3.15” with the desired/latest version. Alternatively, BiocManager can be selected under ‘Packages’ in the bottom right pane of RStudio followed by choosing ‘Update’.

BiocManager::install("DESeq2")

# this installs the DESeq2 package specifically.

Even though these packages have now been installed, they need to be activated each time before use. The *library* command allows these packages to be activated and also confirms the version of these packages that is installed. To verify the version and activate BiocManager, run the following command:

library(BiocManager)

# this will result in an output response in the Console pane (bottom left pane) in RStudio where the exact version of BiocManager will be specified.

# in RStudio, packages can also be activated by selecting them in the bottom right pane, under packages.

library(DESeq2)

# this activates DESeq2.

Now, in order to access and process the gene counts file (featureCounts output that has been saved as a .csv file and has data from different samples compiled together), the directory needs to be set to the folder where the file is saved.

getwd()

# this commands RStudio to verify the current directory.

setwd("/Users/[user name]/RNASeq")

# this command changes the current directory to the folder path indicated. Replace /Users/[user name]/RNASeq with the respective folder path. Note that, unlike Terminal, folder path locations for RStudio require quotation marks.

If the directory has been set to the folder where the gene counts file is located, RStudio can now be prompted to import and process the data in that file. This can be done as follows:

Counts <- read.delim("gene_counts.csv", header=TRUE, row.names = 1, sep = ',')

# ‘gene_counts.csv’ is the name of the file with gene counts compiled from across different samples. ‘Header = true’ indicates that the first row is the column names. ‘Row.names = 1’ indicates that the first column has the identifiers for the rows, in this case the geneIDs. Since the format of the input file is .csv, the separator ‘sep’ can be identified as a comma ‘,’. This step creates a data frame named ‘Counts’ that can then be further manipulated and analyzed. Files can also be imported by navigating to the desired file under ‘Files’ in the bottom right pane in RStudio and choosing ‘Import Dataset’.

Since some genes will have negligible counts/expression, these can be filtered out to avoid errors when running DESeq2. This can be done as follows:

Counts <- Counts[which(rowSums(Counts) > 50), ]

# this further edits the Counts data frame by filtering out the low count genes (keeping only genes with row sums greater than 50).

One thing that can make the analysis easier is to define the experimental conditions by indicating biological replicates of the same experimental group. This can be done by adding a condition vector in R. For instance, in **Figure 3**, it is indicated that there are 12 samples. This is because there are 4 experimental groups, and the experiment was done in triplicate. Samples 1–4 were from the first replicate experiment. Samples 5–8 were from the second replicate experiment. Samples 9–12 were from the third replicate experiment. In other words, Sample 1, Sample 5, and Sample 9 are biological replicates of the same treatment group. Sample 2, Sample 6, and Sample 10 are biological replicates of the same group, and so on.

In R, replicates of the same treatment group can be grouped together. To create this grouping, consider how the samples and replicates are organized in the header of your compiled gene counts file. In our case, there are 4 treatment groups. These will be indicated as S1, S2, S3, and S4. Sample 1, Sample 5, and Sample 9 will be grouped as S1 since these are replicates of that treatment group. Sample 2, Sample 6, and Sample 10 will be grouped as S2, and so on. This can be done by telling RStudio which group (S1, S2, S3, or S4) each column header or sample belongs to. For this particular example, it will look as follows:

condition <- factor(c("S1", "S2", "S3", "S4", "S1", "S2", "S3", "S4", "S1", "S2", "S3", "S4"))

To confirm the group to which each sample was assigned, the command *print(condition)* can be used:

print(condition)

# this will yield an output in the Console pane that shows the group each sample has been assigned to, in the order of column headers listed in the gene counts file.

Next, the sample data needs to be further organized into a data frame. This can be done using the *coldata* command, which combines the column names with the condition information.

coldata <- data.frame(row.names = colnames(Counts), condition)

# this sets the row names of the coldata data frame as the column names of the gene counts file and adds the condition (ie grouping of replicates) we specified previously as a column. Essentially, this is the information about our samples.

coldata

# this prompts RStudio to show the coldata data frame.

Before running DESeq2, a DESeq2 data set needs to be created, as follows:

dds <- DESeqDataSetFromMatrix(countData = Counts, colData = coldata, design = ~condition)

# this creates the DESeq2 dataset.

dds <- DESeq(dds)

# this runs DESeq2 normalization and statistical analysis which may take a few seconds/minutes. The output data object will be saved as ‘dds’ which can be used for further downstream analyses.

To examine the variance in gene expression between different samples, a principle component analysis (PCA) plot can be plotted, which shows how the samples cluster together or apart from another based on gene expression parameters [18]. This essentially serves as a quality assurance step to check for batch effects and to confirm the effect of experimental manipulation. Prior to generating a PCA plot, a variance stabilizing transformation (VST) needs to be performed. This can be run as follows [19]:

vsdata <- vst(dds, blind = FALSE)

# this generates a data object called ‘vsdata’ from the ‘dds’ data object (output of DESeq2).

plotPCA(vsdata, intgroup = "condition")

# this now generates a PCA plot using the variance stabilized data object created earlier (vsdata). Note that the plot will be shown in the ‘Plots’ pane at the bottom right corner of RStudio. This can then be exported into the desired folder. Generally, biological replicates for the same experimental group should cluster together.

Another quality assurance plot that can be generated is a dispersion estimates plot. Dispersion plots illustrate biological variation across replicates [18]. This can be plotted as follows:

plotDispEsts(dds)

## STEP 9: Performing pairwise comparisons between samples in RStudio

Since the aim of many RNA sequencing analyses may be the comparison of gene expression levels from a particular treatment compared to the control (or to another experimental group), the following steps outline how to perform pairwise comparisons between different groups. This will also allow for the generation of volcano plots that compare one group to another as will be shown later. These steps can be repeated for different experimental or control groups as required.

res_S2_vs_S1 <- results(dds, contrast = c("condition", "S2", "S1"))

# in this pairwise comparison, the gene expression levels from DESeq2 output in the S2 condition are being compared to the S1 condition. This creates a data frame called res_S2_vs_S1 that has the genes that show differential expression in replicates in S2 condition compared to replicates in S1 condition.

Next, any genes that have values that could not be computed can be omitted so that genes with differences between the two groups can be computed.

sigs_S2_vs_S1 <- na.omit(res_S2_vs_S1)

# the na.omit() function removes genes with missing values (NA) and creates a data frame titled sigs_S2_vs_S1.

To filter statistically significant differentially expressed genes (DEGs), the false discovery rate (FDR) or adjusted p-value can be set to 0.05 (or lower, as preferred) [20,21]. FDR, instead of p-value, is applied to such data sets since there is a large number of genes involved, which results in a multiple testing problem.

sigs_S2_vs_S1 <- sigs_S2_vs_S1[sigs_S2_vs_S1$padj < 0.05,]

# this sets the FDR (or adjusted p-value) at 0.05 and updates the sigs_S2_vs_S1 data frame to only include the statistically significant DEGs.

This can be repeated as required if performing pairwise comparisons across multiple groups, for example, comparing data from different treatment groups to a control group. This may look like the following:

res_S2_vs_S1 <- results(dds, contrast = c("condition", "S2", "S1"))

res_S3_vs_S1 <- results(dds, contrast = c("condition", "S3", "S1"))

res_S4_vs_S1 <- results(dds, contrast = c("condition", "S4", "S1"))

sigs_S2_vs_S1 <- na.omit(res_S2_vs_S1)

sigs_S3_vs_S1 <- na.omit(res_S3_vs_S1)

sigs_S4_vs_S1 <- na.omit(res_S4_vs_S1)

sigs_S2_vs_S1 <- sigs_S2_vs_S1[sigs_S2_vs_S1$padj < 0.05,]

sigs_S3_vs_S1 <- sigs_S3_vs_S1[sigs_S3_vs_S1$padj < 0.05,]

sigs_S4_vs_S1 <- sigs_S4_vs_S1[sigs_S4_vs_S1$padj < 0.05,]

If such multiple pairwise comparisons are being run, all the data can be compiled into one data frame, which can simplify the plotting of data into volcano plots as will be discussed later on. These data frames can be compiled together using the following sample code:

all_results <- rbind(

 data.frame(Comparison = "S2_vs_S1", sigs_S2_vs_S1),

 data.frame(Comparison = "S3_vs_S1", sigs_S3_vs_S1),

 data.frame(Comparison = "S4_vs_S1", sigs_S4_vs_S1)

)

To add genesymbols to this file, the org.Hs.eg.db package can be used along with the AnnotationDbi [22], which is a Bioconductor package (installed in previous steps). This adds the gene symbol for each ENSEMBL name in a new column. This command is available for a limited set of organisms, e.g., org.Ms.eg.db for mice.

BiocManager::install("org.Hs.eg.db")

library(org.Hs.eg.db)

library(AnnotationDbi)

# A prompt may appear asking if all/some/none of the updates should be installed. For this, an ‘a’ can be typed in the console pane of RStudio which indicates that all updates should be installed.

Convert the data to a data frame:

sigs_S2_vs_S1.df <- as.data.frame(sigs_S2_vs_S1)

# this creates a new data frame called sigs_S2_vs_S1 for the pairwise comparison between S2 and S1 samples

Then map the ENSEMBL IDs to gene symbols:

sigs_S2_vs_S1.df$gene_name <- mapIds(org.Hs.eg.db, keys = rownames(sigs_S2_vs_S1.df), keytype = "ENSEMBL", column = "SYMBOL")

# this adds the column 'gene_name' containing gene names to the sigs_S2_vs_S1.df

An additional column can be added that classifies genes into different categories based on expression (upregulated, downregulated, or not significantly affected):

sigs_S2_vs_S1.df$diffexpressed <- "NO"

sigs_S2_vs_S1.df$diffexpressed[sigs_S2_vs_S1.df$log2FoldChange > 0.6 & sigs_S2_vs_S1.df$pvalue < 0.05] <- "UP"

sigs_S2_vs_S1.df$diffexpressed[sigs_S2_vs_S1.df$log2FoldChange < -0.6 & sigs_S2_vs_S1.df$pvalue < 0.05] <- "DOWN"

This data frame can then be saved as a .csv file with

write.csv(sigs_S2_vs_S1.df, file = "S2_vs_S1_sigs.csv")

## STEP 10: Generating an ordered list of DEGs in RStudio

One of the analyses that could be of interest to researchers aiming to assess the changes to gene expression in response to a particular treatment or in a particular group is to obtain an ordered list of DEGs. Genes must first be sorted according to the magnitude of fold change. Since fold change can be positive representing upregulated genes or negative representing downregulated genes, the absolute values of log2 fold changes can be used to sort genes. Log2 fold change can also be exported in this file of sorted DEGs so that the magnitude and direction (upregulated or downregulated) of fold change for each gene can be assessed. This file containing sorted DEGs and their respective log2 fold changes can be generated as follows:

sigs_S2_vs_S1_sorted <- sigs_S2_vs_S1[order(abs(sigs_S2_vs_S1$log2FoldChange), decreasing = TRUE), ]

# this sorts genes based on absolute values of log2 fold change in descending order.

log2FC_file_S2_vs_S1 <- "S2_vs_S1_DEGs_log2FC.txt"

# since the output file will be exported in the next step, this specifies the file name (“S2_vs_S1_DEGs_log2FC.txt”) for the subsequent step.

write.table(

 data.frame(

 GeneID = rownames(sigs_S2_vs_S1_sorted),

 log2FoldChange = sigs_S2_vs_S1_sorted$log2FoldChange

 ),

 file = log2FC_file_S2_vs_S1,

 sep = "\t",

 quote = FALSE,

 row.names = FALSE,

 col.names = TRUE

)

# this step now exports the data frame with sorted DEGs and includes GeneID and log2FoldChange as rows in this text file. This file will be saved in the working directory under the previously defined name “S2_vs_S1_DEGs_log2FC.txt”.

To also include p-values and adjusted p-values (FDR) in the exported text file along with log2 fold change, the following code can be run subsequently:

pvalue_file_S2_vs_S1 <- "S2_vs_S1_DEGs_pvalues.txt"

# again, the file name is specified ("S2_vs_S1_DEGs_pvalues.txt"), which is now named to include p-values.

write.table(

 data.frame(

 GeneID = rownames(sigs_S2_vs_S1_sorted),

 log2FoldChange = sigs_S2_vs_S1_sorted$log2FoldChange,

 pvalue = sigs_S2_vs_S1_sorted$pvalue,

 padj = sigs_S2_vs_S1_sorted$padj

 ),

 file = pvalue_file_S2_vs_S1,

 sep = "\t",

 quote = FALSE,

 row.names = FALSE,

 col.names = TRUE

)

# this now exports a new file with sorted DEGs that includes GeneID, log2foldchange, pvalue, padj as columns in the text file.

Repeat for any other pairwise comparisons of interest.

These ordered lists can be used to identify and probe DEGs significantly affected and also for further downstream analyses such as examining gene ontology, which can be performed with software such as GOrilla or others.

## STEP 11: Generating heatmaps in RStudio

A potential route for the visualization of differential gene expression patterns is through the generation of heatmaps. Heatmaps cluster genes with similar expression patterns together and also display the phylogenetic relationships between genes. A heatmap that shows expression patterns of DEGs across treatment groups is described here. This heatmap is generated using the *pheatmap* function in RStudio [9].

install.packages("pheatmap")

# the pheatmap function is installed.

library(pheatmap)

# pheatmap function is loaded.

In **step 9**, data frames with pairwise comparisons were created, and the data was further filtered to omit genes with missing values and filtered for an FDR or padj of 0.05. This step created the data frames sigs_S2_vs_S1, sigs_S3_vs_S1, and sigs_S4_vs_S1, which contained significant DEGs. These data frames can be used to plot a heatmap showing patterns of differential gene expression across different treatment groups.

top_genes_S2_vs_S1 <- rownames(sigs_S2_vs_S1)

top_genes_S3_vs_S1 <- rownames(sigs_S3_vs_S1)

top_genes_S4_vs_S1 <- rownames(sigs_S4_vs_S1)

# this code extracts the geneIDs from the data frames generated previously (‘sigs_S2_vs_S1’, ‘sigs_S3_vs_S1’, ‘sigs_S4_vs_S1’) that contained significant DEGs (filtered by FDR at 0.05). A new set of data frames (top_genes_S2_vs_S1, top_genes_S3_vs_S1, top_genes_S4_vs_S1) is created with significant DEGs from each pairwise comparison.

common_top_genes <- Reduce(intersect, list(top_genes_S2_vs_S1, top_genes_S3_vs_S1, top_genes_S4_vs_S1))

# since the heatmap compares gene expression levels across all conditions, common genes that show differential expression in the different pairwise comparisons are identified. This creates a new data frame with the genes that are common across the different pairwise comparisons.

top_counts <- Counts[common_top_genes, c("Sample1", "Sample5", "Sample9", "Sample2", "Sample6", "Sample10", "Sample3", "Sample7", "Sample11", "Sample4", "Sample8", "Sample12")]

# this step filters the original ‘Counts’ data frame which contained gene count data into a new file with gene counts for the common genes, identified in the previous step, only. This step also rearranges the columns to the desired order of samples although this will be specifically performed in the next step.

custom_order <- c("Sample1", "Sample5", "Sample9", "Sample2", "Sample6", "Sample10", "Sample3", "Sample7", "Sample11", "Sample4", "Sample8", "Sample12")

# this code is used to specify the desired order of samples in the heatplot. The goal is to order the treatment groups as S1, S2, S3, and S4. Therefore, the order of replicates (eg Sample 1, Sample 5, and Sample 9 which are S1 condition) is specified.

colnames(top_counts) <- custom_order

pheatmap(top_counts, scale = "row", show_rownames = FALSE, cluster_cols = FALSE,

 annotation_colors = list(Condition = c("Sample1" = "red", "Sample2" = "green", "Sample3" = "blue", "Sample4" = "purple")),

 legend = TRUE, main = "Heatmap of DEGs")

# this code plots the heatmap using the ‘topcounts’ data frame with DEGs that show significant changes across the different treatment groups. The ‘scale = “row”’ part of the code allows the gene expression values to be normalized across samples. ‘Show_rownames’ is set to false so that the GeneIDs are not listed which would otherwise create a lot of clutter. ‘cluster_cols = false’ is also used so that samples (columns in the ‘topcounts’ data frame) are not clustered and rather arranged by the desired order described earlier. Other design parameters are specified such as color, a legend is added and ‘main’ specifies the title of the plot.

png(file = "heatmap_pairwiseDEGs.png", width = 1600, height = 1600, units = "px", res = 300)

pheatmap(top_counts, scale = "row", show_rownames = FALSE, cluster_cols = FALSE,

 annotation_colors = list(Condition = c("Sample1" = "red", "Sample2" = "green", "Sample3" = "blue", "Sample4" = "purple")),

 legend = TRUE, main = "Heatmap of DEGs")

dev.off()

# this code exports the plot (Figure 4) saving it as a .png image file in the working directory with the colors as indicated.

**Figure 4.**
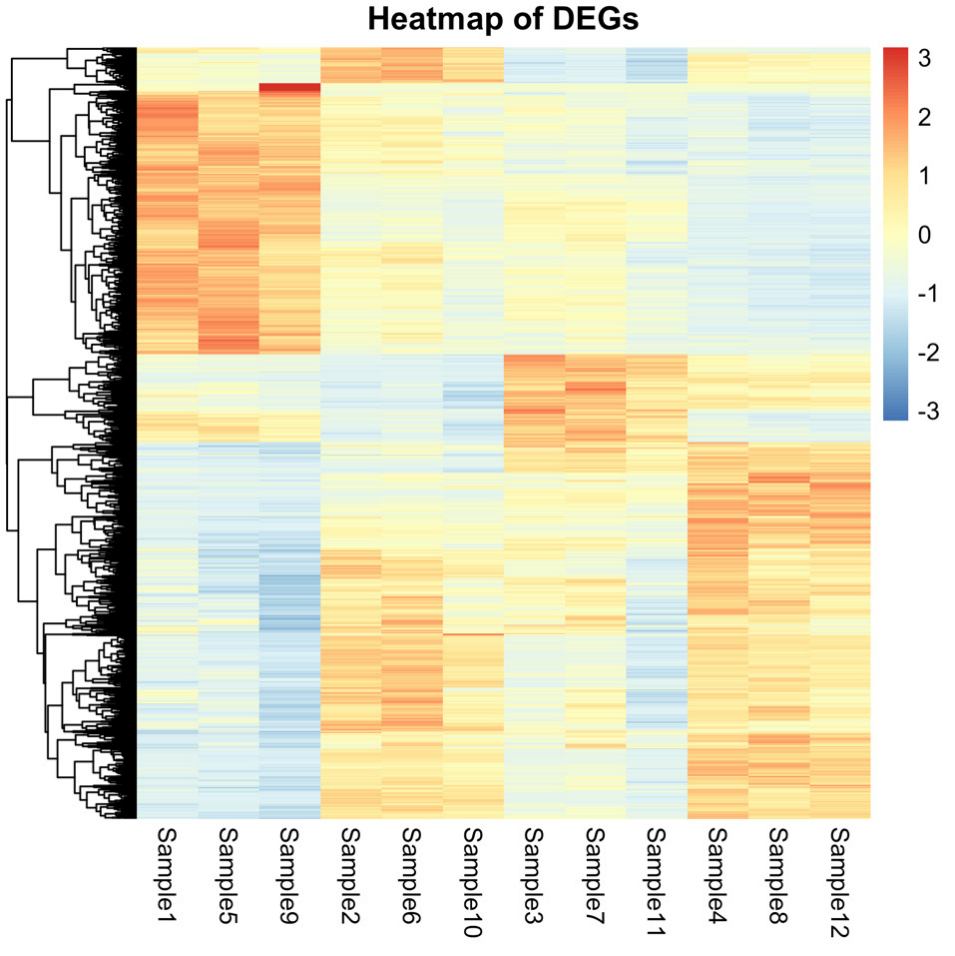
Heatmap generated in step 11 displaying patterns of gene expression across samples. Samples are listed in the desired order so that the first three columns represent S1 condition, the next three columns represent S2 condition, and so on. The scale on the right side represents gene Z-scores and ranges from -3 (most prominently downregulated genes) shown in blue to +3 (most prominently upregulated genes) shown in red.

## STEP 12: Generating volcano plots in RStudio

Typical analysis of DEGs involves a graphical representation of the specific genes that are upregulated, the genes that are downregulated, and those that are not significantly affected in a given pairwise comparison between others. This can be achieved by plotting on a volcano plot that shows significantly upregulated genes on the right, significantly downregulated genes on the left, and genes that are not significantly affected (below the 0.05 FDR cutoff) in the middle. Volcano plots can be generated using the *ggplot2* [10] and *ggrepel* [11] functions in RStudio.

install.packages("ggplot2")

install.packages("ggrepel")

library(ggplot2)

library(ggrepel)

# this installs and loads ggplot2 and ggrepel.

**Figure 5.**
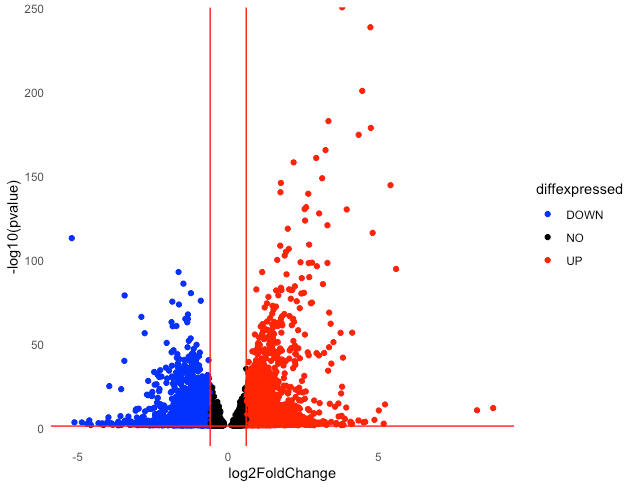
Basic volcano plot. Downregulated DEGs are shown in blue, upregulated DEGs in red, and non-significant genes in black for the S2 group compared to the S1 group.

First, a simple plot without gene labels can be generated. This will show the distribution of DEGs and non-significant genes in a given sample compared to another. To do so, the pairwise comparisons previously generated in **Step 9** that were compiled into one large data frame titled “all_results” can be used. A volcano plot showing DEGs in the S2 group compared to the S1 group can be plotted as follows **(Figure 5)**:

diff_S2_vs_S1 <- data.frame( log2FoldChange = sigs_S2_vs_S1$log2FoldChange, pvalue = sigs_S2_vs_S1$pvalue, padj = sigs_S2_vs_S1$padj, gene_id = rownames(sigs_S2_vs_S1))

# this extracts the relevant columns for the S2 vs S1 pairwise comparison specifically from the data frame with all compiled pairwise comparisons (all_results).

diff_S2_vs_S1$diffexpressed <- "NO"

diff_S2_vs_S1$diffexpressed[diff_S2_vs_S1$log2FoldChange > 0.6 &

 diff_S2_vs_S1$pvalue < 0.05] <- "UP"

diff_S2_vs_S1$diffexpressed[diff_S2_vs_S1$log2FoldChange < -0.6 &

 diff_S2_vs_S1$pvalue < 0.05] <- "DOWN"

# these three codes categorize genes based on their p-value and fold changes into non-significantly affected genes, upregulated genes, or downregulated genes, respectively.

p <- ggplot(data = diff_S2_vs_S1, aes(x = log2FoldChange, y = -log10(pvalue), col = diffexpressed)) +

 geom_point() +

 scale_color_manual(values = c("NO" = "black", "UP" = "red", "DOWN" = "blue")) +

 theme_minimal() +

 geom_vline(xintercept = c(-0.6, 0.6), col = "red") +

 geom_hline(yintercept = -log10(0.05), col = "red") +

 theme(panel.grid = element_blank())

# the ggplot function is used to plot DEGs from the S2 vs S1 pairwise comparison in a volcano plot. The x-axis represents log2 of the fold change (hence, left is downregulated and right is upregulated). The Y-axis represents log10 of the p-value. Geom_point describes the data points on the plot. Col = diffexpressed specifies that the color of data points will be based on the expression categories (upregulated, downregulated or not significantly affected) specified in the previous code. These colors are further specified to be black for non-significant genes, red for upregulated DEGs and blue for downregulated DEGs. Further graphing details are specified such as plotting a red horizontal line (geom_hline) representing the 0.05 FDR cut off line and two vertical lines (geom_vline) at -0.6 and 0.6 log2 fold change representing -1.5 and 1.5 fold change in expression, respectively. A white background for the plot with no gridlines is specified using theme_minimal and theme(panel.grid = element_blank(), respectively.

ggsave("basic_volcano_plot.png", plot = p, width = 6, height = 4, dpi = 300)

# this exports the plot (Figure 5) to the working directory saving it as a file titled ‘basic_volcano_plot’ at the specified dimensions.

**Figure 6.**
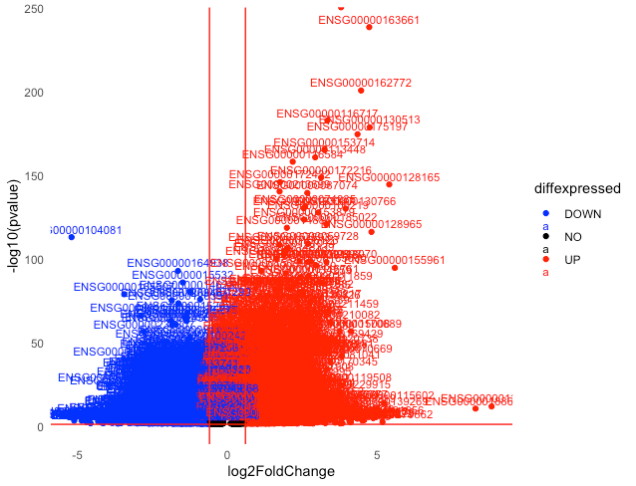
Volcano plot with labeled DEGs. Downregulated DEGs are shown in blue, and upregulated DEGs are shown in red for the S2 group compared to the S1 group. Data points for non-significant genes (in black) are not labeled.

To examine which particular genes are affected, gene labels can be added **(Figure 6)**. This can be done as follows:

diff_S2_vs_S1$delabel <- ifelse(

 !is.na(diff_S2_vs_S1$gene_id) & diff_S2_vs_S1$diffexpressed != "NO",

 diff_S2_vs_S1$gene_id,

 NA

)

# this code uses a conditional function (ifelse) to assign gene labels to upregulated and downregulated DEGs. Only genes that do not have missing geneIDs and that are significantly differentially expressed are selected for labelling.

p <- ggplot(data = diff_S2_vs_S1, aes(x = log2FoldChange, y = -log10(pvalue), col = diffexpressed)) +

 geom_point() +

 scale_color_manual(values = c("NO" = "black", "UP" = "red", "DOWN" = "blue")) +

 geom_text(aes(label = delabel), vjust = -0.5, size = 3) +

 theme_minimal() +

 geom_vline(xintercept = c(-0.6, 0.6), col = "red") +

 geom_hline(yintercept = -log10(0.05), col = "red") +

 theme(panel.grid = element_blank())

# the plot is created again with the gene labels now over the data points.

ggsave("labelled_volcano_plot.png", plot = p, width = 6, height = 4, dpi = 300)

# this saves the plot (Figure 6) in the working directory at the specified dimensions as a file titled ‘labelled_volcano_plot.png’.

As shown in **Figure 6**, labeling all genes produces a practically illegible plot. This highlights the need to be able to observe genes in smaller sets at a time. One such analysis is the gene set enrichment analysis (GSEA), which examines how genes within a particular gene set are affected; for example, only those genes relevant to a particular biological process or disease. This interrogation of specific gene sets of particular interest to the scope of research can be visualized using volcano plots that plot all DEGs but only generate gene labels that belong to a particular gene set. Gene sets of interest to the researcher can be pre-selected and obtained from the Molecular Signature Database (MSigDB):

https://www.gsea-msigdb.org/gsea/msigdb/human/genesets.jsp.

To generate a volcano plot with only genes from a specific gene set labeled, the focus is essentially the overlap of genes between genes 1) that are significantly differentially upregulated/downregulated (i.e., significant DEGs) and 2) genes that comprise a given gene set of interest.

Since genes within gene sets are listed as gene names rather than gene IDs, gene IDs will need to be converted into gene names so that overlap can be detected. Additionally, converting gene IDs to gene names will make visualization on the plot easier, as gene names are shorter and allow for easier identification of which genes are upregulated and downregulated.

Converting gene IDs to gene names was already performed earlier in **step 9** using Org.Hs.eg.db and AnnotationDbi packages (Option #1) or can alternatively be performed manually (Option #2).

Note that the exported file from **step 9** (S2_vs_S1_sigs.csv) is more relevant than the ordered lists generated in **step 10**, as these lists lacked the categorization of genes as upregulated, downregulated, or not significantly affected, which is required for the generation of volcano plots.

‘Gene ID’ to ‘gene name’ conversion:

Option #1:

In **step 9**, a file called “S2_vs_S1_sigs.csv” was exported, which contained a column titled “gene_name” and a column titled “diffexpressed,” which categorized genes into upregulated, downregulated, or not significantly affected. This file can be imported again into R so that it can be used to generate volcano plots. This can be done as follows:

diff_S2_vs_S1_gnames <- read.csv("S2_vs_S1_sigs.csv")

Option #2:

Alternatively, gene names can be added manually to a file that contains a column with ENSEMBL gene IDs. To do this, a file with the genes can be exported as follows:

write.csv(diff_S2_vs_S1, "diff_S2_vs_S1.csv", row.names = FALSE)

# this exports the diff_S2_vs_S1 data frame which has genes categorized into upregulated, downregulated or non-significantly differentially expressed genes.

# to manually convert gene IDs, the diff_S2_vs_S1 data frame file can be opened (eg using Excel) once exported and the entirety of the geneIDs column can be copied. This column can then be pasted into a geneID converter (such as https://www.biotools.fr/human/ensembl_symbol_converter) to convert ENSEMBL IDs into gene names. Note that the column header (gene_id) should be deleted once results are pasted before converting. It is also recommended to uncheck the ‘keep original IDs in output’ option so that only the gene names are produced as an output which can be copied for pasting as a single column in excel. These gene names can be pasted into a new column in excel in the diff_S2_vs_S1 file with the column labelled gene_names.

# this file with gene names added can now be saved as diff_S2_vs_S1_gnames in a .csv format.

Back in R, this file with gene names for DEGs can now be imported:

diff_S2_vs_S1_gnames <- read.csv("diff_S2_vs_S1_gnames.csv")

# this imports the file.

diff_S2_vs_S1_gnames$diffexpressed <- "NO"

diff_S2_vs_S1_gnames$diffexpressed[diff_S2_vs_S1_gnames$log2FoldChange > 0.6 & diff_S2_vs_S1_gnames$padj < 0.05] <- "UP"

diff_S2_vs_S1_gnames$diffexpressed[diff_S2_vs_S1_gnames$log2FoldChange < -0.6 & diff_S2_vs_S1_gnames$padj < 0.05] <- "DOWN"

# this further modifies the diff_S2_vs_S1 data frame (which now also includes gene names) by categorizing genes into upregulated, downregulated and non-significantly affected genes.

Now, the gene set of interest can be obtained and downloaded from MSigDB. For instance, this analysis will look at the GOBP_GOLGI_VESICLE_TRANSPORT gene set [gene ontology biological process (GOBP) with 307 genes known to be involved in Golgi vesicle transport].

# to obtain the list of genes, the ‘further investigate the X number of genes’ button on the page for each gene set can be pressed to obtain a list of the gene names within the gene set.

# gene names (titled ‘Input Gene Identifiers’) that comprise a particular set can be copied and pasted into a new excel file that can be imported into R. The excel file will have just one column containing all the genes that can also be titled ‘gene_name’ to keep the notation the same as in the diff_S2_vs_S1_gnames data frame. For the GOBP_GOLGI_VESICLE_TRANSPORT gene set described here, the file with gene names is saved as golgi_vesicle_genes in a .csv format for ease.

Back in R, this file with gene names from the gene set can now be imported:

genes_to_label <- read.csv("golgi_vesicle_genes.csv", header = TRUE)

# this imports the ‘gene_name’ column within the golgi_vesicle_genes file and creates a data frame called ‘genes_to_label’. This data frame now contains the gene names from the gene set of interest.

Next, the overlap of significant DEGs with genes from the gene set of interest (imported in the previous step) needs to be determined.

significant_genes <- subset(diff_S2_vs_S1_gnames, diffexpressed %in% c("UP", "DOWN"))

# as described earlier, genes have been previously assigned to non-significantly affected genes, upregulated genes and downregulated genes. These categories can be used to exclude non-significant genes and create a data frame with significant DEGs. This code creates a data frame from the file with gene names titled ‘significant_genes’ containing only significantly upregulated and downregulated genes.

genes_to_label <- subset(genes_to_label, gene_name %in% significant_genes$gene_name)

# the ‘genes_to_label’ data frame is further edited to only include genes (or more specifically, gene names) that are significantly differentially expressed (upregulated and downregulated) from the significant_genes data frame. Therefore, genes that will be labelled on the plot must be both differentially expressed and within the selected gene set of interest.

**Figure 7.**
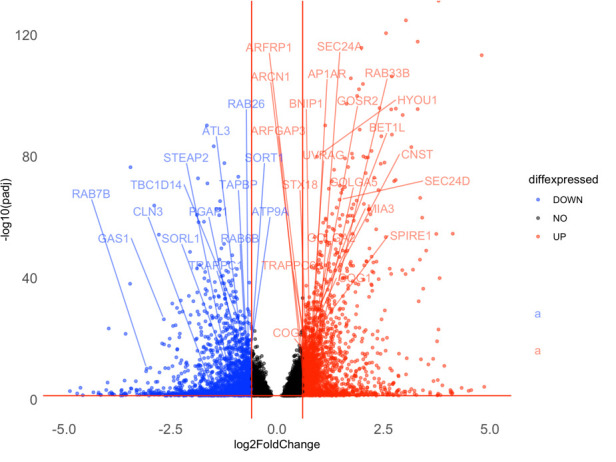
Volcano plot with a selected set of DEGs labeled. Downregulated genes are shown in blue, and upregulated genes are shown in red in the S2 group compared to the S1 group. Only genes that are both significant DEGs and that are present in a pre-selected gene set of interest (GOBP_GOLGI_VESICLE_TRANSPORT) are labeled.

Finally, this volcano plot (with DEGs that belong to the labeled gene set of interest) can be plotted as follows **(Figure 7)**:

p <- ggplot(data = diff_S2_vs_S1_gnames, aes(x = log2FoldChange, y = -log10(padj), col = diffexpressed)) +

 geom_point(size = 1) +

 theme_minimal() +

 geom_text_repel(

 data = subset(diff_S2_vs_S1_gnames, gene_name %in% genes_to_label$gene_name & diffexpressed != "NO"),

 aes(x = log2FoldChange, y = -log10(padj), label = gene_name),

 box.padding = 0.6,

 point.padding = 0.3,

 max.overlaps = 20,

 min.segment.length = 0,

 nudge_y = 5.5,

 vjust = -15.3

 ) +

 scale_color_manual(values = alpha(c("blue", "black", "red"), alpha = 0.5)) +

 geom_vline(xintercept = c(-0.6, 0.6), col = "red") +

 geom_hline(yintercept = -log10(0.05), col = "red") +

 theme(panel.grid = element_blank(),

 axis.text.x = element_text(size = 13),

 axis.text.y = element_text(size = 13)) +

 ylim(0, 125) +

 xlim(-5, 5)

# this code plots all genes in the pairwise comparison of S2 compared to S1 but only significant DEGs that are upregulated or downregulated and that are present in the specified gene set of interest (GOBP_GOLGI_VESICLE_TRANSPORT) are labelled. Since larger gene sets can include many more genes, many of the parameters relevant to labels can be adjusted which include box.padding, point.padding, max.overlaps, nude_y, vjust..etc.

ggsave(filename = "S2_vs_S1_golgivesicle_plot.png", plot = p, width = 8, height = 6, dpi = 300)

# this saves the plot (Figure 7) in the working directory at the specified dimensions as a file titled ‘S2_vs_S1_golgivesicle_plot.png’.

This volcano plot **(Figure 7)** not only represents a more manageable representation of data but the overall method also allows researchers to investigate perturbations to specific gene sets of interest and graphically represent such an analysis.

Alternatively, another thorough approach that allows for interrogating activation of specific gene sets and for generating GSEA plots with enrichment score (using the gene counts file generated in **step 7**) can be performed by GSEA software available to download here:

https://www.gsea-msigdb.org/gsea/index.jsp.

This can be repeated for other pairwise comparisons and/or for other gene sets of interest to the researcher.

**Concluding remarks**

As the utilization of genomics technology, particularly RNA-Seq, to interrogate genome-wide changes becomes more widespread, beginner-friendly analysis approaches are required, as many researchers may have limited familiarity with the analysis and may not have access to bioinformaticians. In this paper, a computational pipeline is described that allows the identification and representation of transcriptomic changes in response to different treatment groups. First, gene count information is obtained by performing quality control analysis on the read data, trimming off adapters and low-quality reads, mapping the mRNA reads to the genome, and counting the number of reads per gene. These steps are carried out in Terminal or command line, ideally in a high-performance Linux computer. Next, differential gene expression analysis is performed in RStudio using the *DESeq2* package. Further data visualization methods such as heatmaps and volcano plots that allow the analysis of differential expression patterns or the specific probing of gene expression changes within particular gene sets, respectively, are also described. The outcome of RNA-Seq analysis by these methods is the identification of novel gene families that drive the biological processes or disease under interrogation. Note that an example analysis using data from the GEO is provided as Supplementary Information.

## Validation of protocol

This protocol has been used and validated in the following research article:

Shouib et al. [2]. Inflammatory gene regulation by Cdc42 in airway epithelial cells. Cellular Signalling

Throughout this protocol, result figures are based on the data used for the publication above.

## Supplementary information

The following supporting information can be downloaded here:

1. Shouib 2025 Supplemental Data
